# Provider Perspectives on Sexual Health Services Used by Bangladeshi Women with mHealth Digital Approach: A Qualitative Study

**DOI:** 10.3390/ijerph17176195

**Published:** 2020-08-26

**Authors:** Yamin Tauseef Jahangir, Amit Arora, Pranee Liamputtong, Mohammad Hayatun Nabi, Samantha B. Meyer

**Affiliations:** 1 School of Public Health and Health Systems, University of Waterloo, Waterloo, ON N2L 3G1, Canada; ytjahangir@uwaterloo.ca (Y.T.J.); samantha.meyer@uwaterloo.ca (S.B.M.); 2 Department of Public Health, School of Health and Life Sciences, North South University, Dhaka 1229, Bangladesh; hayat4008@yahoo.com; 3 School of Health Sciences, Western Sydney University, Campbelltown, NSW 2560, Australia; p.liamputtong@westernsydney.edu.au; 4 Translational Health Research Institute, Western Sydney University, Campbelltown, NSW 2560, Australia; 5 Oral Health Services, Sydney Local Health District and Sydney Dental Hospital, Surry Hills, NSW 2010, Australia; 6 Discipline of Child and Adolescent Health, Sydney Medical School, Faculty of Medicine and Health, The University of Sydney, Westmead, NSW 2145, Australia

**Keywords:** sexual health, mHealth, STI service, sexual healthcare, access to healthcare, Bangladesh

## Abstract

Cases of sexually transmitted infections (STIs) are underreported in Bangladesh. Women in general suffer from poor sexual health outcomes due to a lack of access to sexual health services. mHealth, a digital approach to STI services, is an easier and cheaper way to disseminate health information in Bangladesh. However, women have less autonomy in accessing STI services and it is important to learn if, how and/or why women use mHealth. A qualitative study was conducted with 26 medical doctors to explore their perceptions of the mHealth STI services used by Bangladeshi women. Themes were grouped under four categories: (1) provider perceptions of mHealth for sexual healthcare; (2) the health literacy of women clients; (3) cost and maintaining timeliness in providing mHealth services; (4) mHealth service accessibility. Data suggest that mHealth can play a significant role in improving the awareness and utilization of STI services in Bangladeshi women. Successful opportunities for STI service expansion using mHealth were identified, depending on the quality and type of service delivery options, awareness of challenges related to health literacy framework, cost, accessibility to information and availability of culturally competent health experts to disseminate health information. We identify the need to increase access and use of mHealth services for sexual health, as it provides an innovative platform to bridge the health communication gaps in sexual health for Bangladeshi women.

## 1. Introduction

Although 61% of married women in Bangladesh use contraceptive methods, the prevalence of contraceptive use is lower in rural areas than in urban Bangladesh [1,2]. Additionally, according to the Bangladesh Demographic Health Survey (2000), sexually transmitted infection (STI) cases in particular, are often underestimated among sexually active women, as many of the STI symptoms are not easily recognized and many STIs do not have visible symptoms [2]. Bangladesh, being a developing country, faces challenges in reporting STIs as elaborate laboratory methods are scarce [1]. In developing regions each year, an estimated 204 million women have one of the four major curable STIs (chlamydia, gonorrhea, syphilis or trichomoniasis), but 170 million (82%) do not receive sexual health services [3]. Bangladeshi women have been documented to be vulnerable to STIs, including HIV/AIDS, and their knowledge about different diseases is reported to be poor [4]. Studies report that 40% of ever-married women had never heard of HIV/AIDS, 19% had heard but did not know how to prevent it and only 41% knew ways to prevent disease transmission [5]. Therefore, although research suggests that among married women with no education, 87% have refused sex with their husband if they knew their partner had an STI [2], STIs remain a problem as cases are often not identified, and therefore remain undiagnosed.

STIs are an important public health problem, negatively impacting the health, social life, and economic situation of women. Negative consequences for health include not only physical discomfort but also infertility, ectopic pregnancies, cervical cancer, fetal wastage, low birth weight, infant blindness, neonatal pneumonia and neurological or cognitive damage to the fetus [6]. Socially, a lack of awareness and cultural taboos increase a woman’s risk of contracting STIs as a consequence of stigmatization in the community and associated non-disclosure of STIs. Also, as a result of stigma, women may be less likely to discuss their sexual health problems and seek appropriate treatment [4].

Despite the acknowledgement that there is a growing need to raise awareness about how to reduce rates and manage STIs in Bangladesh, patients diagnosed with STIs receive very limited or no counselling on safe sex, HIV transmission, and partner referral, largely because of time constraints of the service providers [7]. In Bangladesh, there are limited reports on STI cases for women. However, a descriptive retrospective cross sectional study between 2003-2011 on 30,152 patients (72% males and 28% females) found 19.5% females suffered from discharging STIs (e.g., Gonorrhea, Vulvovaginal candidiasis or VVC), 5.71% from ulcerated STIs (e.g., Syphilis, Chancroid, Genital Herpes), 3.48% had suffered from viral STIs (e.g., Genital Warts, HIV) and 0.98% had Genital Scabies [8]. Moreover, it is suggested that many women face barriers towards sexual health service use due to: poor access to health facilities and related transportation costs; high fees for services; poor quality of clinical services and infection control; insufficient provider training; gender based stigma in communities and among families; provider discrimination against low income women; and a shortage of providers in the health sector [9,10]. 

Bangladesh is one of the most densely populated developing countries globally, and there remains a huge disparity in health care distribution between rural and urban locations [9]. Such disparities in developing countries often extend to ethnicity and education as well, and regional demographics add further to these disparities [4,9,10]. For example, rural people suffer from poorer health outcomes due to lack of medical expertise and health care facilities in their regions [10]. Mobile health or ‘mHealth’ has been identified as an easier and cheaper way to disseminate healthcare information to the rural areas [2,11,12]. mHealth has been used to overcome widespread health system barriers such as health professional shortages, reliance on untrained and/or informal providers, costs of service and transportation, and lack of reliable sources of health information in developing countries [12].

With respect to mHealth and women’s sexual health, the need for mobile phone technology has been widespread among marginalized populations. mHealth is a tool that can be used to connect women to care and reduce the response time for various sexual health emergencies [13]. There are more than 20 mHealth services currently available in Bangladesh [14]. In some of these mHealth services clients subscribe to a mobile health company supported health service and can dial hotline numbers to speak to medical doctors [15]. In recent times, the mobile phone market has grown in Bangladesh with the latest statistics showing they have more than doubled from 32% in 2007 to 78% in 2011 and then increased up to 89% in 2014 [16]. In 2018, there were 147 million active mobile phone subscribers and a calculated 13.8% annual growth rate in mobile internet usage in Bangladesh [17,18]. This increase is particularly remarkable in urban areas, where 89% of all household own mobile phones compared with 75% of rural households [17]. Traditional gender roles result in women still lagging behind men in the utilization of sexual healthcare services, and mHealth use in particular may be lower for women because of gender differentials in mobile phone ownership and usage [19]. Indeed, it has been found that women’s limited access to family income has led to lower levels of mobile phone ownership among women. This is particularly pronounced in lower income households [20]. 

mHealth interventions may provide a way to improve access to, and awareness and use of sexual health services for Bangladeshi women. mHealth may therefore be a cost-effective and innovative means of bridging the sexual healthcare service gap. However, there remains a paucity of research on how and if women use mHealth services for sexual health. Therefore, the aim of our study was to explore healthcare provider perceptions of mHealth for sexual health services by Bangladeshi women. The ultimate goal is to assist policymakers, health care providers, and relevant stakeholder organizations working on mHealth to strengthen sexual health service provision for women in Bangladesh.

## 2. Materials and Methods 

Interviews with healthcare providers were conducted at a non-government organization (NGO) located in Dhaka, Bangladesh. Given the limited number of mHealth organizations that provide sexual health services, both public and non-government organizations were contacted for data collection. From the seven organizations contacted, only one non-government organization offering mHealth services, located in the urban location at Mohakhali, Dhaka showed interest and gave their informed consent for this study. As it was deemed important to understand the perspectives of healthcare providers and their experiences towards barriers or factors to mHealth service use by women, we proceeded with recruitment through this NGO. The mHealth service organization in this study covers all 64 districts of the country using a mobile operator company network. The organization has 55 medical doctors to serve on all medical consultation services, while 35 dedicated medical doctors are available to provide sexual health related information to clients. The mobile health services include doctor consultation, medical directory services which provide 24/7 phone directory services for hospitals, doctors, diagnostic centres and ambulance service. 

In line with the aim of the research, participants were purposively recruited [21,22]. Participants included health service providers with certified medical degree such as, medical doctors; health service providers with sexual health/STI care management experience; health service providers having prior training using mHealth technology such as operating mobile apps/hotline numbers/attending client calls. It was important to include these various roles to allow us to understand the broader decision-making process by women clients regarding their use or not of mHealth and specifically: the barriers and/or facilitators towards accessing sexual health services, and how sexual health or STI care is understood or negotiated within local knowledge and through mHealth systems. Of the 35 doctors contacted, a total of 26 doctors offering sexual health services were interviewed. There were 14 male and 12 female medical doctors. Of those interviewed, almost all participants had 7–8 years of work experience and the majority had been working with mHealth services for more than one year. Participant age ranged from 30–50 years and the majority completed their medical degrees from public medical colleges and hospitals. All participants resided in urban locations in Dhaka city. The 26 interviews conducted allowed us to meet data saturation, as little new data emerged towards the completion of data collection [23,24,25,26,27]. 

As these participants worked on roster duties, interviews were conducted after office hours and at convenient locations identified by participants (e.g., café/restaurant or household) between June to September 2019. Ethics approval was obtained from the Department of Public Health, North South University, Bangladesh. Informed consent was obtained from all individual participants included in the study. The research team developed semi-structured interview guides based upon our knowledge of the literature and gaps this research was designed to interrogate, with questions reflecting the areas of sexual health, knowledge on sexual health and practices, sexual health service utilization or treatment-seeking behaviour of women for sexually transmitted infections, and patient preferences and perceptions of sexual health services in Bangladesh (e.g., STI service). Interviews were recorded and identification numbers were assigned to each recording in order to protect confidentiality. Recordings were later used to create complete transcripts in the native language of Bangla, which were then translated verbatim into English and reviewed (transcripts were checked against the tapes for accuracy) [25,26]. The translation process from Bangla to English was overseen by senior researchers to ensure the translations retained the information from the verbal account and were true to the original data [26]. Data were analyzed using the thematic guidelines of Braun and Clarke (2006) [26]. This process includes open coding, recoding, creating categories and abstraction [24,25]. The statements relevant to the objectives of our study were marked, numbered, coded in the transcript in each interview, and then organized into conceptually relevant categories for the exploration of patterns and themes using NVivo Pro 12 [26,27,28]. The end result is a thematic summary that provides a rich nuanced report and a strong inductive qualitative reasoning [26,27,28]. A subset of the research team met on a weekly basis to agree on the coding and categorization. When the categorization was completed, the research team examined the categories for conceptual overlapping and for the emerging themes, while any discrepancies in coding and themes allowed the team to review all the collated extracts to check how they form the coherent pattern. However, since thematic analysis requires a considerable amount of freedom in analyzing data, the authors maintained relative flexibility and did not focus entirely on the prevalence of a theme, but rather on the meaningful information that related to the overall research question. The transcripts were checked to ensure that no relevant content was overlooked. The co-authors discussed on the analysis and interpretation of the data until consensus was reached.

## 3. Results

Our study identified themes that were grouped into four categories, namely: (1) provider perception of mHealth for sexual healthcare; (2) health literacy of women clients; (3) costing and maintaining timeliness in providing mHealth services; (4) mHealth service accessibility.

### 3.1. Provider Perception of mHealth for Sexual Healthcare

mHealth services sought and provided were identified as ranging from the general disease inquiry, sexual and reproductive health factors such as menstruation, sexual health complications such as STIs, learning about various kinds of STIs, reproductive tract infection information, general STI information, fixing appointment dates for diagnosis with nearby clinics, counselling on STI complications, verbal diagnosis on pregnancy complications, and nutrition profiles for pregnant mothers. Eighteen participants mentioned that they received more calls regarding STIs from women than from men, while these participants mentioned that the majority of the complications were related to syphilis and genital warts.

“I give advice over the phone. Patients over phone ask about different problems, what to do, where to go, etc. I provide all the relevant information on sexual and reproductive health and also on STI cases. I suggest them for inquiring to our other diagnostic services as well, whenever I see their symptoms suggest more (clinical diagnosis) medical checkup. I suggest the pregnant mothers to take proper diet, to take nutritious food, and to take enough rest. I tell them to go regularly for checkup.” (MD, 1 year experience)

Participants had varied perceptions about their role in providing mHealth. Eighteen participants said that mHealth services are quite good in providing counselling to clients with STI complications, while eight remarked that it was not always possible to provide diagnoses of STIs over the phone, and that a physical examination is necessary. In most cases, participants said that female clients called to inquire about generic reproductive information. Ten providers mentioned “helplines” available for clients to call to obtain health-related services. Some participants mentioned that clients were made aware about the customer care, mobile retailer centres, recharge points, audio and visual media.

“If you ask me, I would say this is a good service system that is now working and making some progress in Bangladesh. The counselling process for women’s health can allow them to get quick information, and clarify any urgent matter.” (MD, 1 year experience)

“I don’t think so. If conversations were enough then physical checkup wouldn’t be needed. There are differences between physical check-ups and mobile phone conversations. There is a huge gap between these. For example, if anyone says that she is having STI complications then we will not understand the reason just by hearing her over the phone. Moreover, we do understand the nature of stigma attached in a patriarchal society. But even then, I think a lot of women suffer more because they delay in the decision-making process for a proper diagnosis.” (MD, 2 years experience)

“There are helplines available for health related information. We often tell women to call in helpline numbers if they are in need for more information. Those services can relate to generic health information or can provide address or phone number for nearby clinics or diagnostic centres offering sexual health services like STI diagnosis.” (MD, 3 years experience)

When asked about the benefits of mHealth services, all the participants mentioned that it made women feel optimistic about their service centre that provides a wide range of options, starting from ambulance contact service to receiving discounts for some specific diagnostic centres. Participants mentioned that women clients sometimes also talk about their family members. Therefore, the program not only focuses on women’s reproductive health from a household perspective, but is more holistic, as it is an information dissemination platform whereby information can flow through women. This in itself was very helpful for women who in most cases felt reluctant in acting as agents of their own healthcare in a male dominated household.

“Women clients do talk about other problems of their family members as well, especially of their daughters. They often want to know about hygiene practices and on any sexual health complications. If you ask me, then I think mHealth acts as a platform for women to get information on both generic health information to particular information about STI illness.” (MD, 2 years experience)

### 3.2. Health Literacy of Women Clients

Low levels of literacy, and poor health literacy, were noted as barriers to care, and to clients understanding of how to identify STIs, when to seek care, or how to prevent transmission. For example, participants said that some women were often confused about the difference between STIs and STDs and also did not understand the issues of white discharge and what it actually meant for their sexual health. Furthermore, twenty participants mentioned that clients may be well aware of their services but are very cautious about seeking care. Healthcare provider perceptions focused on the fact that clients only call to seek information when there is something ‘wrong’ regarding their health, not taking advantage of preventative services. In a quote from one of the providers:

“Some women are often not sure about STIs and STDs and would want to know. We tell them. They also don’t understand about menstruation regulation, have fear of not understanding what white discharge mean. But you see, women only contact us when they face any problem or are suffering from some symptom or illness for long. Then I can only say, people seek help, when they know there is something wrong…when something is just not right!” (MD, 7 months in service)

According to our participants, health literacy relates not only to how clients understand information, but also how they utilize health information. To address issues related to health literacy, some of the participants reported that they call their clients to check in on whether they have understood the health information properly. However, although a follow-up process exists, it is not a standard practice and at times is limited to special cases (e.g., first trimester complications, severe STI infections etc.). If they did call patients to assess literacy, the medical doctors said that they wanted to determine of women (a) were able to describe the health issue that they encountered and (b) understood the response from the healthcare provider. Further, in the follow-up process, the service providers worked to understand how clients perceived their health conditions by asking questions like “how are you feeling today? Can you explain your health condition today?”, and to asking questions such as “from our last conversation, can you tell me what are the steps you have taken so far for your illness?” Participants said that they spent time explaining medical terms in the simplest non-medical language to ensure that the clients actively understood the health information and made informed choices. Health literacy was identified as a challenge given the short allotted time providers are given in consultations. One participant mentioned:

“If I ask you to take a pill, or go for a referral, will you do it? Probably yes, because you understand what I am talking about. But…(pause), it is hard to describe how women understand our health information. Sometimes they will contact us immediately whenever they face some problem and even follow up with our medical suggestions. Other times they keep their sufferings to themselves and for long, which makes matters worse. You see, if you don’t do what we recommend, that surely means you are showing either ignorance to your own health and well-being, or you did not understand what we said. That’s why we follow up; sometimes it is challenging to learn that they prefer to listen to people who are not experts in health, and not us! It makes things rather difficult to make them (women clients) understand about all the complications so that they prioritize on their health.” (MD, 3 years experience)

### 3.3. Cost and Maintaining Timeliness in Providing mHealth Services

All participants noted that clients have to undergo a simple mobile registration process to get access to mHealth services. After the registration is complete, it is the duty of the medical doctor at the centre to make a phone call to the client initially and provide all the necessary information about the mHealth program. The participants mentioned that this service allows the clients to feel content and be in touch with them regarding accessing the services in their community. The client completes the initial registration to the system and then the person pays a service fee of BDT 28 (0.35 USD) for the first consultation. The minimal charge of BDT 2 (0.025 USD) was identified as a factor that was beneficial for improving access and use of mHealth services.

Participants said that mHealth is also effective in terms of time management for women. Twenty of the participants said that from the clients’ point of view, mHealth saves their time (e.g., compared to visiting hospitals that are far away or any other private formal healthcare practitioner) and also provides timely health information. Some participants noted that in times of crisis, the service is a good way to give valuable and timely information to the clients, for example, regarding first trimester complications, STI complications, menstruation problems, during labour pain problems, and nutritional facts during gestational age.

“The process saves time. It is effective in terms of addressing critical conditions of our clients. I think sometimes it so happens that they call even from remote locations and so I give them the right suggestion. I fear for their well-being. I want that in any complication the patient could be brought to the hospital through a phone call. So, we even connect with our nearby hospitals to get in touch with such clients to manage the situation. This is very helpful.” (MD, 2 years experience)

Providers also noted that timely treatment is beneficial for client outcomes, decreasing mortality rates. All participants mentioned that it is their duty to serve the clients at all times so the clients should not feel hesitant to seek care. However, the management of client call flow was noted as a challenge for providers. Participants mentioned that there is a waiting time for each call that can be long if there is a high number of calls. It was suggested that a high number of calls suggests that mHealth services are necessary and being used, but that time management is a difficult task.

“At times I cannot manage patient flow, there are some waiting. Clients often keep calling back and I try to answer to all queries. It is what I am supposed to do. But it is challenging as some callers can take longer time than others.” (MD, 1 year experience)

### 3.4. mHealth Service Accessibility

Twenty participants said that mHealth services are effective in terms of providing greater access to health information for women regarding STIs. However, eight also emphasized that effectiveness of the service related to STI cannot be improved solely only through mHealth. They said that greater access through alternate means is still required, for example through short messaging service (SMS). At the time of data collection SMS was limited to basic information in Bangla texts that focused on emphasizing on the importance of consuming essential nutrients and about immunization, but not STIs. Additionally, participants noted that the social stigma and conservativeness attached to the concept of STIs is a challenge in providing SMS information as not all information can be covered within a short text. Fourteen participants mentioned that the clients were often concerned about confidentiality and that once they were assured that privacy is maintained in the consultation session, they were more likely to share information about their health needs.

“Access to information is an important thing. If you do not provide the right platform for information accessibility, then it means clients will not confide in your service in future. We have a text messaging service system. We think this is also effective in allowing our clients to access health information. But we do not have it exclusively for sexual and reproductive health purpose, such as, in terms of infectious diseases. But in a conservative society like ours, the question is how far can you achieve this with SMS? Well, it is an area, we need to think about, on how to create or maintain a proper continuity in access to information.” (MD, 2 years experience)

## 4. Discussion

In Bangladesh, considerable steps have been taken to organize a complex array of health information systems that underpin the overall management of the health sector. For example, in 2016, Bangladesh created the standardized local electronic health bulletin for easy access of health facility data [29]. Bangladesh also formed the Bangladesh workforce strategy prioritizing five major areas namely: planning and development; distribution; retention and professional engagement; performance standards; and information systems. Related to these priorities, in 2011, Bangladesh was one of the 15 countries that use mHealth to raise health awareness while in 2016 the country continued to invest in mHealth [12,29,30]. It is important to now understand how and if mHealth services are used to improve population health and in the case of our research, sexual health for Bangladeshi women. Therefore, the aim of our study was to explore healthcare provider perceptions of mHealth for sexual health services used by Bangladeshi women.

Study findings in Chakaria Upazilla of Bangladesh [19] found that only 1% of the community reported to know about the “helpline” service options and that the community lacked basic understanding of mHealth services. However, our findings indicate that women are aware of and using mHealth sexual health services and that providers receive calls from all over Bangladesh. A possible reason for this awareness could be socio economic condition, social support, prior experiences with the broader healthcare system, familiarity with the mHealth program and cultural influence—all of which may allow clients to access mHealth services [31]. However, previous research suggests that the usability and user acceptance of mHealth is a challenge in both urban and rural locations, as government service providers have better knowledge on eHealth and mHealth systems, while service recipients are often not well equipped with mHealth knowledge [31]. Although a reason could be for poor functional literacy rate (45.3%) and geographical location in rural areas, the people of Bangladesh mostly rely on low-tech Information, Communication and Technology (ICT) options to access health related information [32]. 

Our data also suggest that interventions that build community capacity and empower women, particularly around literacy, are needed to improve sexual health more generally. Findings are consistent with previous studies [32] suggesting that the majority of clients were illiterate, leading to challenges for the use of sexual health information being disseminated through mHealth services [33]. However, through this study, we learned about patient sexual health literacy from the lens of providers—how these providers experienced the perspectives of their clients in retaining and utilizing sexual health information. Thus, the concept of health literacy was more from the provider’s point of view, which studies have shown there may remain differences from the way laypeople and health professionals understand and defined health [34]. This has been explained as a problem related to cultural competence in service delivery. Stereotyped “do’s” and “don’ts” (e.g., in medication management, infection treatment, diagnostics) constructed by healthcare providers can often cause patients to be defined by a fixed set of ethnic or cultural attributes [35]. This in turn may cause variations in how patients understand and perceive certain health information. Therefore, the involvement of ethnically, linguistically and culturally congruent health care providers to work with patients may be needed in order to acknowledge the diversity of patient experiences and improve sexual health literacy amongst Bangladeshi women clients so that, sexual health services, and mHealth specifically, may have a greater impact for this population. 

Improved accessibility was identified as a benefit of mHealth services. However, existing literature suggests that there may be some barriers to implementing such technology on a larger scale. For example, administrative staff in the hospital sector are at times reluctant to make changes to new technology and implement new ideas [36]. Such behaviour has been described in another study as a ‘resistance of change’ [37], as staffs consider such training have flaws in the implementation level. On the contrary, the medical doctors in our study were willing to perform their duties with mHealth technology, were committed to their profession, and were open to new technological developments to ensure their clients had proper accessibility to sexual health information. Doctors were optimistic about SMS technology, too, although issues regarding the sensitivity of sexual health remained a concern. 

In a study on the urban slum community setting noted that the community often used mobile phones to call and secure appointments with doctors [14]. Our study identified a much broader use of mobile phones and suggests that mobile health applications that go beyond service bookings to actually provide access to sexual health information is promising for women in our research. Our findings suggest that most clients used mHealth services for generic health and frequently for STI related information. Even in a more conservative society, women still opted to talk more freely with health care providers using mHealth services especially on sexual health and STI care and would seek proper counselling and advise. Therefore, our study showed that mHealth service systems can be a reliable framework for providing sexual health services and any future health interventions should use a women-centred approach. However, as noted earlier, women are 21% less likely to own a mobile phone than men and this gender disadvantage is the highest in South Asia, followed by sub-Saharan Africa [38]. Furthermore, married women are suggested to be restricted in the use of mobile phones and ability to seek health information as a consequence of men’s preferences and men’s control over women’s mobiles and finances [39]. Hence, while our data suggest that mHealth is used by women, it is important to consider subsets of the population who face additional barriers related to accessing the technology necessary to use such services. It will be critical to determine who cannot benefit from such technologies and ensure that alternative resources are still available and widely promoted. 

Our findings identified that doctors view mHealth services as being important for addressing challenges in sexual health service use related to distance from healthcare centres, time and cost. While there are subscription costs involved, they were deemed minimal and were not perceived to be a barrier to sexual health service use. These data are consistent with existing research identifying that participants largely use mHealth because it is cost effective, time efficient, provides instant treatment and requires no transportation costs [19].

In terms of limitations, our data only reflect formal healthcare providers’ perspectives. It was beyond the aim of the research to obtain consumer or client perspectives, which would allow us to identify women’s perceptions of the perceived benefits, challenges, costing and overall accessibility of mHealth services. Additionally, as a consequence of our sampling approach, there is a risk of social desirability biases [40]. This may have occurred as these providers are clearly invested in mHealth and may benefit from the affirmation of the benefit of services. 

## 5. Conclusions

Our study explored medical doctors’ perceptions of mHealth for sexual health provision by Bangladeshi women. We found that mHealth can play a significant role in women’s sexual health and overall well-being. Successful opportunities for STI service expansion with mHealth were identified and depend on the quality and type of service delivery options, awareness of challenges related to health literacy, cost, accessibility of information and availability of culturally competent health experts. This study identified a need to increase access to and use of mHealth services for sexual health, as it provides an innovative platform to bridge the health communication gaps in sexual health for Bangladeshi women. In light of these findings, it is important to realize that mHealth approach is in its inception and yet creating more proving grounds as an innovative platform in Bangladesh to address critical healthcare information, for example, on sexual health in general and STI in particular. Steps should be taken in terms of increasing medical practitioner recruitment, providing training opportunities on cultural competence, learning more about the missing links of health literacy and culturally relevant sexual healthcare models to address issues of stigma, poor understanding of STI risk and related physical harms.
